# Polarization Raman Imaging of Organic Monolayer Islands for Crystal Orientation Analysis

**DOI:** 10.1021/acsomega.0c06313

**Published:** 2021-03-31

**Authors:** Toki Moriyama, Takayuki Umakoshi, Yoshiaki Hattori, Koki Taguchi, Prabhat Verma, Masatoshi Kitamura

**Affiliations:** † Department of Applied Physics, Osaka University, 2-1, Yamadaoka, Suita, Osaka 565-0871, Japan; ‡ PRESTO, Japan Science and Technology Agency, 4-1-8 Honcho, Kawaguchi, Saitama 332-0012, Japan; § Department of Electrical and Electronic Engineering, Kobe University, 1-1, Rokkodai-cho, Nada, Kobe 657-8501, Japan

## Abstract

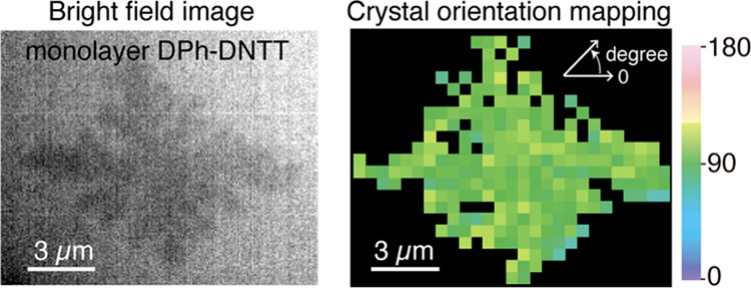

An organic semiconductor film made of diphenyl derivative dinaphtho[2,3-*b*:2′,3′-*f*]thieno[3,2-*b*]thiophene (DPh-DNTT) has high carrier mobility. However, this mobility may be greatly affected by the crystal orientation of the DPh-DNTT’s first layer. Polarization Raman microscopy is widely used to quantitatively analyze the molecular orientation, and thus holds great potential as a powerful tool to investigate the crystal orientation of monolayer DPh-DNTT with high spatial resolution. In this study, we demonstrate polarization Raman imaging of monolayer DPh-DNTT islands for crystal orientation analysis. We found that the DPh-DNTT sample indicated a strong dependence of the Raman intensity on the incident polarization direction. Based on the polarization dependence, we developed an analytical method of determining the crystal orientation of the monolayer DPh-DNTT islands and experimentally confirmed that our technique was highly effective at imaging the islands’ crystal orientation with a spatial resolution of a few hundred nanometers.

## 1 Introduction

Thin-film organic semiconductors have been widely studied for their high applicability originating from their lightweight, flexibility, and low processing cost. Organic field-effect transistors (OFETs),^1^ organic photovoltaics,^2^ and organic light-emitting diodes^3^ are well recognized as these semiconductors’ primary applications. With the advancement of organic electronics, the development of organic semiconducting materials has garnered significant attention, among which dinaphtho[2,3-*b*:2′,3′-*f*]thieno[3,2-*b*]thiophene (DNTT) is one of the most promising because of its high stability in air conditions and high carrier mobility.^4−6^

In DNTT-based OFETs, a thin DNTT film as a channel layer is usually deposited on a substrate with a gate insulator via vacuum evaporation, and carrier transport occurs within a few nanometers of the film from the film/gate interface.^7^ Therefore, the physical properties, e.g., morphology and crystal orientation, of the organic film at the initial stage of film growth should be understood, as they significantly affect the carrier mobility.^8−11^ Regarding film morphology, atomic force microscopy (AFM) and scanning electron microscopy are often utilized^12,13^ because they provide detailed information on nanoscale morphological features. To investigate the crystal orientation of thin films, X-ray diffraction^14−20^ and low-energy electron diffraction^21−23^ are the common techniques. However, they usually offer spatially averaged information, making it difficult to understand the spatial variation of the crystal orientation.

Recently, our group has reported that polarized light microscopy enables crystal orientation analysis with sub-micrometer spatial resolution,^24^ suggesting that such optical techniques are promising for such analysis at a high spatial resolution. Polarization Raman microscopy is widely recognized as a powerful, noninvasive method often used for molecular orientation analysis,^25−31^ and it also provides the chemical information of a sample. Therefore, it holds great potential as an effective tool for studying the crystal orientation of the initial stage of DNTT layer growth at a high spatial resolution.

In this study, we demonstrate polarization Raman microscopic imaging of monolayer diphenyl derivative DNTT (DPh-DNTT) islands for crystal orientation analysis. As part of the DNTT series, DPh-DNTT has high thermal stability and is hardly affected by the device structure, including a bottom gate on the rough substrate.^32,33^ As the DPh-DNTT film comprises a multilayered structure, it forms monolayer islands in the initial stage of the growth process. Herein, we fabricate monolayer DPh-DNTT islands through vacuum evaporation and confirm that they exhibit several Raman peaks corresponding to the vibrational modes of the DPh-DNTT molecule. Raman signals from organic molecule monolayers are usually very weak, so several techniques are sometimes utilized for signal enhancement, such as the resonant Raman effect^34^ and plasmonic enhancement.^35,36^ However, we realized that these techniques are not very suitable for our purpose. The resonant excitation resulted in strong photoluminescence from DPh-DNTT. Locating a metal near the sample for plasmonic enhancement affected the structure of the DPh-DNTT sample. Therefore, we precisely optimized our homemade Raman microscope to maximize the collection efficiency of Raman signals and achieved Raman measurements of the monolayer DPh-DNTT islands with normal Raman spectroscopy. Thus, there was no need to use these enhancement techniques in this study. Through polarization-dependent Raman measurements, we reveal that the Raman peak intensities are highly sensitive to the incident polarization. Therefore, we develop an analytical method to image the islands’ crystal orientation based on the correlation between the incident polarization and intensity of a Raman peak. Consequently, we demonstrate that polarization Raman imaging enables crystal orientation mapping of monolayer DPh-DNTT islands at approximately 300 nm resolution.

## 2 Results and Discussion

**Figure 1:**
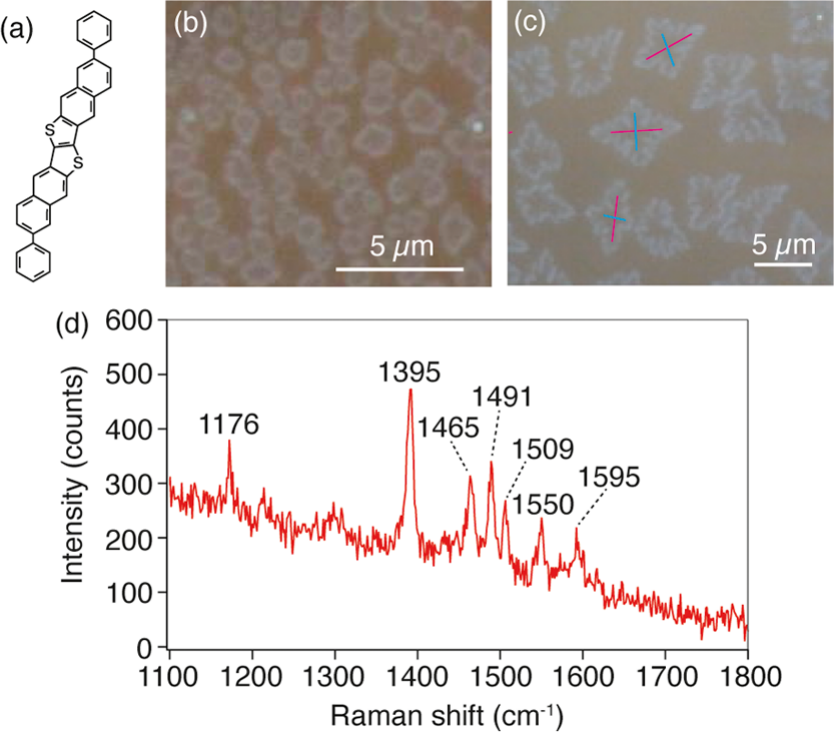
(a) Chemical structure of DPh-DNTT molecules. (b) Dark-field optical image of round DPh-DNTT islands. (c) Dark-field optical image of cruciform DPh-DNTT islands. (d) Raman spectrum of DPh-DNTT (laser power 240 μW, exposure time 5 s, excitation wavelength 532 nm).

A Si wafer on which a SiO_2_ layer was thermally grown was used as a substrate. We deposited DPh-DNTT on the substrate by vacuum deposition to form monolayer islands. Figure 1a shows the chemical structure of a DPh-DNTT molecule. In monolayer DPh-DNTT islands, the molecules stand almost vertically to the substrate, forming a herringbone structure. We fabricated the monolayer DPh-DNTT islands in two different shapes, i.e., round and cruciform, which were controlled by the substrate temperature during the deposition as well as prior substrate treatments. The round islands were deposited at 160 °C by vacuum deposition on a substrate treated with UV–O_3_. Meanwhile, the cruciform islands were deposited at 185 °C on a substrate treated by HF solution. Figure 1b,c shows dark-field optical microscopy images of the round and cruciform islands, respectively. As indicated by the pink and blue lines in some of the cruciform islands in Figure 1c, these islands typically exhibited long and short axes. The pink lines correspond to the long axis, and the blue lines correspond to the short axis. We confirmed via AFM that the islands were monolayer (Figure S1). Their thickness was approximately 2.3 nm, nearly the length of a DPh-DNTT molecule itself.

For the investigation of the islands, we used a homemade Raman microscope. In the setup, a backscattering configuration was applied. A single-mode excitation laser (wavelength: 532 nm) was tightly focused on the sample plane through an objective lens (Nikon, 150×), which has a high numerical aperture (NA) of 0.95 for better spatial resolution as well as better collection efficiency. The focal spot size is estimated to be around 350 nm, which is the diffraction-limited size. The incident light passed through a half-wave plate and a polarizer so that we could control the polarization direction of the incident light. The scattered light was collected by the same objective lens. The scattered signals were then introduced into a spectrometer through a depolarizer and detected by a Peltier-cooled CCD camera (PIXIS:100BRX, Teledyne Inc.). A slit was set at the entrance of the spectroscope to eliminate background signals generated at locations away from the incident focal spot. The slit width was set to ∼100 μm. A lens was placed before the slit to focus Raman signals on the slit, the focal length of which was 60 mm. We placed as few mirrors and lenses in the detection path as possible to maximize the efficiency of signal collection by avoiding unwanted reflection losses at the surface of each optical component. A piezo scanner (Piezoconcept Inc.) was installed on the sample stage and synchronized with the CCD camera for Raman imaging. A highly stable sample stage (KS-N, Nikon Inc.) was utilized for long-term Raman imaging. More details are provided in our previous report.^26^

We evaluated the Raman spectrum of the round DPh-DNTT islands. Several peaks originating from the molecular vibrations of DPh-DNTT were observed in the spectrum, as shown in Figure 1d, corresponding to our previous report.^30^ The background signal that appeared in the spectrum is probably a weak tail of the luminescence signal from the sample. Previous reports showed that compounds of the DNTT series usually have an optical band gap at a wavelength of around 450 nm.^37−39^ Although the excitation wavelength of 532 nm is slightly out of the absorption peak, it can still excite weak photoluminescence from DPh-DNTT, which is not a problem for obtaining Raman signals. We also confirmed that when the excitation wavelength of 442 nm was used, strong photoluminescence was observed from DPh-DNTT, which overwhelmed Raman signals as shown in Figure S2a. This also indicates that the DPh-DNTT has the band gap at around 450 nm.

**Figure 2:**
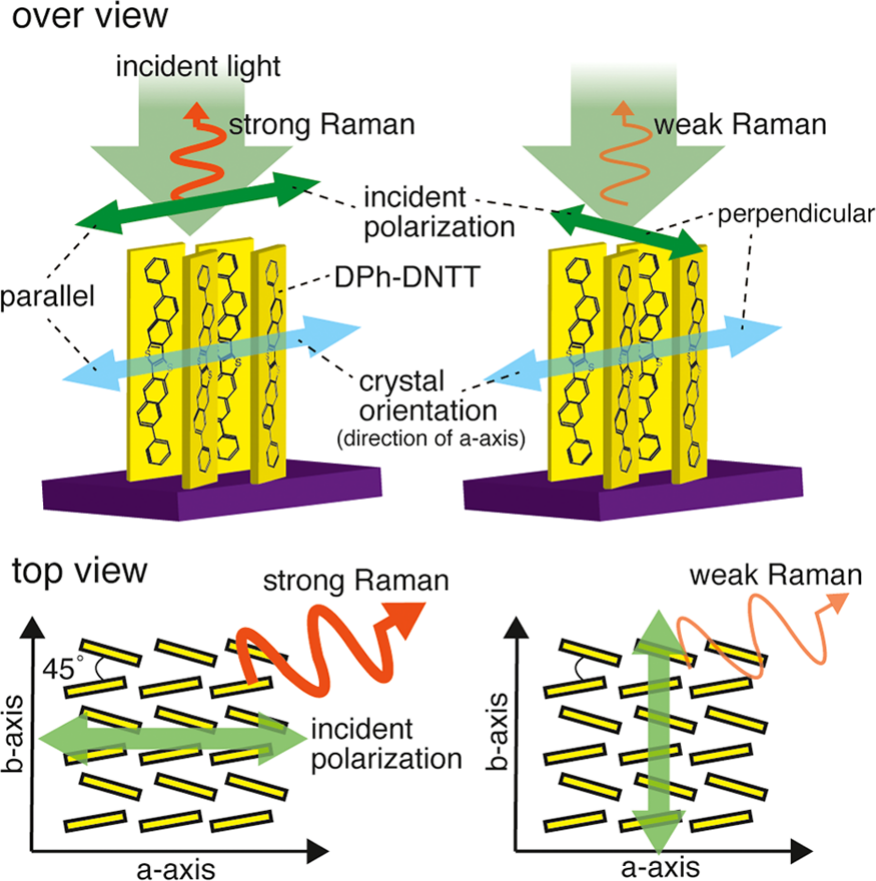
Schematic illustration of the relationship between the polarization of incident light and crystal orientation of the DPh-DNTT molecules. The molecules stood vertically on a substrate and formed a herringbone structure. Strong Raman scattering was expected when the incident polarization was parallel to the crystal orientation (*a*-axis direction).

According to our previous study, molecular vibration modes of DPh-DNTT oscillate in the in-plane direction of the molecule.^30^ Therefore, we expected a strong Raman intensity in the case of incident polarization parallel to the molecular plane and a weak Raman intensity in the case of incident polarization perpendicular to the molecular plane. In an actual situation, DPh-DNTT forms a herringbone structure as illustrated in Figure 2, wherein the herringbone angle of the DPh-DNTT is approximately 45°.^40^ Therefore, one can expect a strong Raman intensity when the incident polarization is parallel to the *a*-axis of the herringbone structure and a weak Raman intensity when the incident polarization is perpendicular to the *a*-axis, as depicted in Figure 2.

**Figure 3:**
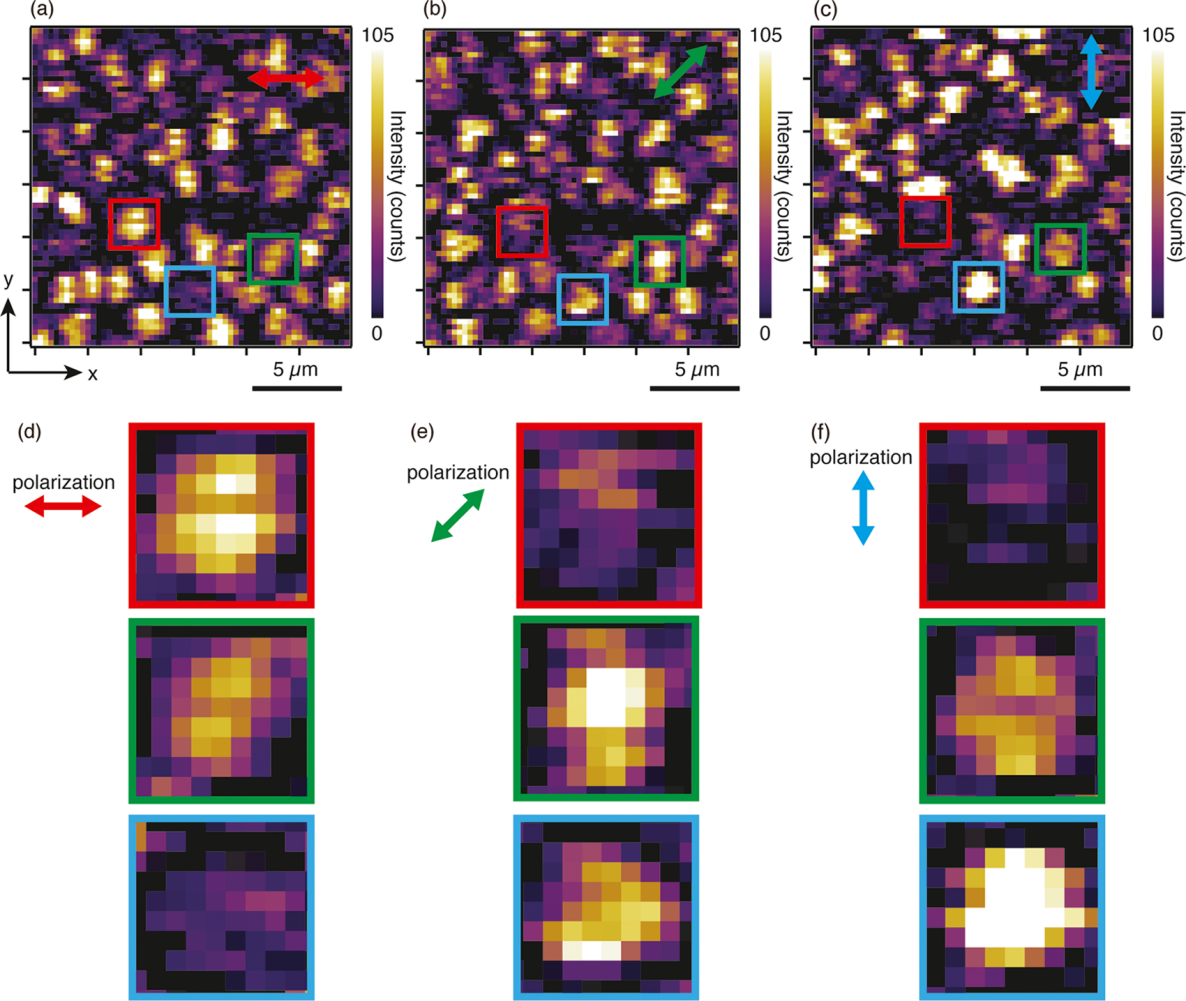
(a–c) Raman images of round DPh-DNTT islands in the same area with different polarization angles (laser power 240 μW, exposure time 2 s, excitation wavelength 532 nm). The polarization directions are indicated by the colored arrows in each image. (d) Zoomed-in images of the DPh-DNTT islands indicated by the red, green, and blue squares in (a). (e) Zoomed-in images of the DPh-DNTT islands indicated by the red, green, and blue squares in (b). (f) Zoomed-in images of the DPh-DNTT islands indicated by the red, green, and blue squares in (c).

To investigate whether DPh-DNTT islands show such polarization dependence, we demonstrated Raman imaging of our round DPh-DNTT islands with different incident polarizations. Figure 3a–c shows three Raman images taken in the same area with different incident polarization directions. In Figure 3a, we used the polarization direction parallel to the *x*-axis in the image, and we rotated it by 45° in Figure 3b. The polarization direction parallel to the *y*-axis is applied in Figure 3c. We used the Raman peak at 1395 cm^–1^ to construct Raman intensity images, as it yielded the strongest Raman intensity compared with the other peaks. We applied Lorentzian curve fitting to the peak to extract intensity counts of the Raman signal only (see the details in Section S3 in the Supporting Information), and the intensity counts were plotted in the images. The laser power was 240 μW in the sample plane (2.62 mW/μm^2^), and the exposure time was 2 s for each pixel. Since we perform Raman imaging multiple times at the same area, it is crucial to avoid sample degradation due to a possible heating effect by laser irradiation to properly investigate the polarization dependence. As shown in Figures S2b and S4, we carefully investigated the sample damage due to laser irradiation and concluded that the laser power lower than approximately 500 μW (5.46 mW/μm^2^) did not induce noticeable sample damage. As shown in Figure 3a–c, since each image showed totally different patterns, we found that the polarization direction affected the Raman scattering intensity from the monolayer DPh-DNTT. To observe the polarization effect more clearly, as shown in Figure 3d, we obtained extended images of some of the individual DPh-DNTT islands from Figure 3a indicated by colored squares. Similarly, we obtained extended images of the same DPh-DNTT islands in the different polarization conditions from Figure 3b,c, as shown in Figure 3e,f, respectively. Each island provided strong Raman intensity when a specific polarization direction was applied. For example, the DPh-DNTT island, surrounded by the red square, had the strongest intensity when the polarization direction was horizontal. Therefore, we expected that the *a*-axis of this DPh-DNTT island was oriented in the horizontal direction. Similarly, the *a*-axis of the island marked by the green square should have been oriented toward the direction of 45°, and the *a*-axis of the island indicated by the blue square should have been oriented toward the vertical direction in the images. Thus, this polarization dependence could be effectively used to quantitatively evaluate the crystal orientation in the monolayer DPh-DNTT islands.

**Figure 4:**
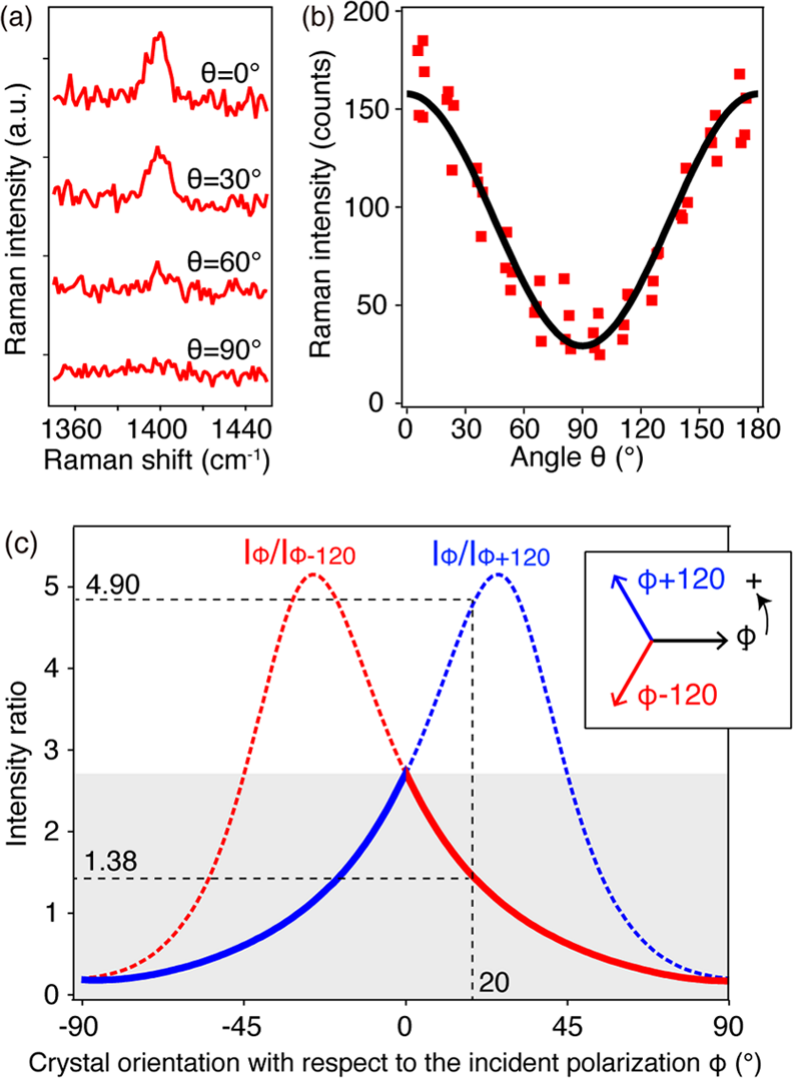
(a) Raman spectra of DPh-DNTT islands obtained in different polarization directions (laser power 240 μW, exposure time 15 s, excitation wavelength 532 nm); θ = 0° corresponds to the incident polarization angle that provided the strongest intensity. (b) Relationship between the incident polarization angle θ and intensity of the Raman peak at 1395 cm^–1^. The black line shows the fitted curve. (c) Relationship between the crystal orientation with respect to a certain incident polarization angle φ and the ratio of Raman intensities obtained at different polarizations. We have set an arbitrary incident polarization angle φ to generalize the relationship, whereas in the case of the incident polarization angle θ, θ = 0° is defined as the angle where the Raman intensity was the strongest. Three polarization angles, φ, φ + 120, and φ – 120, were considered, and two Raman intensity ratios, *I*_φ_/*I*_φ + 120_ and *I*_φ_/*I*_φ_ _– 120_, were calculated. The three arrows in the inset represent the relationship of the three polarization angles. The red curve represents the intensity ratio *I*_φ_/*I*_φ+120_, and the blue curve represents the intensity ratio *I*_φ_/*I*_φ_ _– 120_ with respect to the crystal orientation angle from φ.

As we observed a clear polarization dependence, we precisely investigated it for quantitative analysis. Figure 4a shows the Raman spectra obtained from a DPh-DNTT island in different polarization directions. The vibration mode at 1395 cm^–1^, which was used for analyzing the polarization dependence, is estimated through density functional theory. It mainly shows C–H bending and C–C stretching of aromatic rings, vibrating in the in-plane direction of the DPh-DNTT molecule, as shown in Figure S5. θ represents the incident polarization angle. We defined θ = 0° as the angle that provided the strongest Raman intensity and rotated the polarization angle every 30–90°. The Raman signal became weaker as the polarization was rotated and almost vanished at 90°. Therefore, we measured the Raman spectra by changing the incident polarization angle more precisely. We obtained spectra from several DPh-DNTT islands and plotted the Raman intensity with respect to the polarization angle (Figure 4b), which was well fitted with the function *I*(θ) = *A* + *B* cos^2^ θ. Here, the values of *A* and *B* were optimized through the best fitting, as 27.2 and 128.6, respectively. From this polarization angle dependence, we established a method to quantitatively analyze the crystal orientation, i.e., the direction of the *a*-axis, based on the relationship shown in Figure 4c. When we used two mutually orthogonal polarization directions, uniquely determining the crystal orientation was not possible. Therefore, we applied three different polarizations in this method. We have set an arbitrary incident polarization angle φ to generalize the relationship shown in Figure 4c, whereas we defined θ = 0° as the angle where the Raman intensity was the strongest. We started with the polarization angle φ to obtain the Raman spectrum of a DPh-DNTT island and then obtained spectra at the polarization angles φ + 120 and φ – 120. We thus calculated the Raman intensity ratios *I*_φ_/*I*_φ + 120_ and *I*_φ_/*I*_φ_ _– 120_, where *I*_φ_, *I*_φ + 120_, and *I*_φ_ _– 120_ indicate the Raman intensities obtained at the angles of φ, φ + 120, and φ – 120, respectively. Figure 4c exhibits the relationship between these Raman intensity ratios and the crystal orientation angle with respect to angle φ, which was calculated from Figure 4b. The detailed equations to derive this relationship are described in Section S6 in the Supporting Information. The blue curve represents the intensity ratio *I*_φ_/*I*_φ + 120_, and the red curve represents *I*_φ_/*I*_φ_ _– 120_. To determine the crystal orientation from Figure 4c, we compared the values of *I*_φ_/*I*_φ + 120_ and *I*_φ_/*I*_φ_ _– 120_. When *I*_φ_/*I*_φ + 120_ was smaller than *I*_φ_/*I*_φ_ _– 120_, we could identify the crystal orientation angle with respect to angle φ using the solid part of the blue curve (*I*_φ_/*I*_φ + 120_) in Figure 4c. By contrast, when *I*_φ_/*I*_φ_ _– 120_ was smaller than *I*_φ_/*I*_φ + 120_, the solid part of the red curve (*I*_φ_/*I*_φ_ _– 120_) was used to determine the crystal orientation. For instance, if *I*_φ_/*I*_φ + 120_ = 4.90 and *I*_φ_/*I*_φ – 120_ = 1.38, the solid curve of *I*_φ_/*I*_φ_ _– 120_, ranging from 0 to 90°, would be used, as *I*_φ_/*I*_φ_ _– 120_ is smaller than *I*_φ_/*I*_φ + 120_. Thus, we could determine that the crystal orientation was rotated 20° from the initial polarization angle, φ. Through this analytical procedure, we quantitatively investigated the crystal orientation of the DPh-DNTT islands.

**Figure 5:**
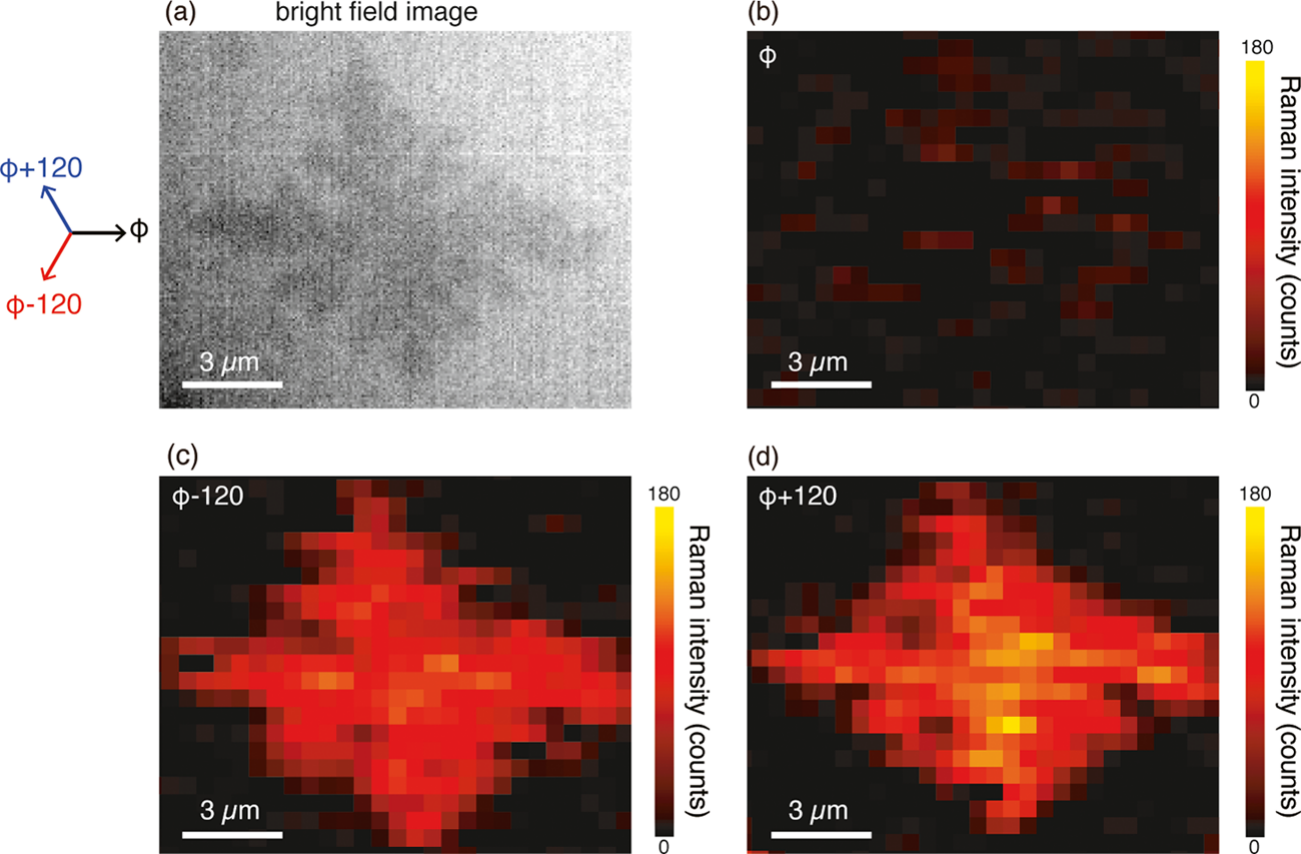
(a) Bright-field optical microscopy image of a cruciform DPh-DNTT island. The arrows indicate the polarization directions used for the Raman imaging. (b–d) Raman images of the island in (a), constructed by the intensity of the Raman peak at 1395 cm^–1^ at the incident polarization angles of φ, φ – 120, and φ + 120, respectively (laser power 420 μW, exposure time 6 s, excitation wavelength 532 nm).

**Figure 6:**
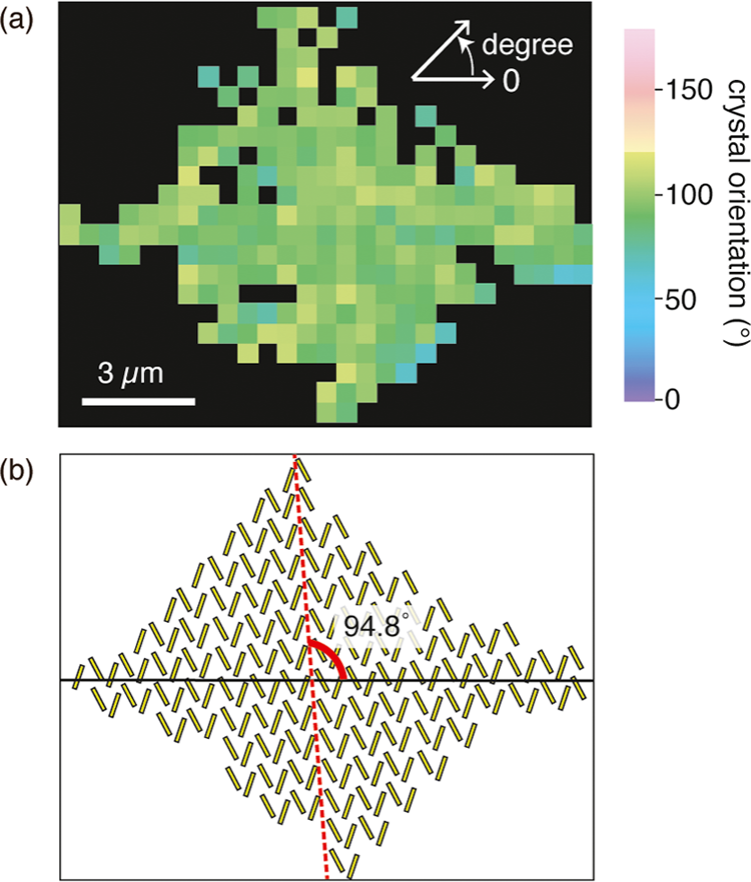
(a) Crystal orientation mapping of the cruciform DPh-DNTT island shown in Figure 5. (b) Schematic illustration of the herringbone structure of the island in (a). The crystal orientation angle (*a*-axis direction) from the horizontal axis is 94.8.

To confirm the validity of our method, we applied it to the analysis of the crystal orientation of a cruciform DPh-DNTT island. As our previous study revealed that the short axis of cruciform DPh-DNTT islands nearly corresponds to the *a*-axis, i.e., the crystal orientation, this was a suitable sample to verify whether our method works properly. Figure 5a shows a bright-field microscopy image of the cruciform island used for our evaluation. Because dark-field observation was not available in our Raman microscopy setup, we used the bright-field configuration. It was still possible to observe the monolayer DPh-DNTT islands in the bright-field images, although the image contrast was decreased. To analyze the crystal orientation angle of the cruciform DPh-DNTT island, we conducted Raman imaging three times at the different polarization angles of φ, φ + 120, and φ – 120, as indicated in Figure 5a. Figure 5b–d shows the three Raman images constructed by the intensity of the Raman peak at 1395 cm^–1^ at the polarization angles of φ, φ + 120, and φ – 120, respectively. As clearly shown, there was a difference in intensity because of the polarization dependence. For angle φ, the Raman intensity was much weaker compared with that of the other cases, probably because the direction of the angle was perpendicular to the short axis, i.e., *a*-axis, of the cruciform island. After taking the images, we obtained the intensity ratio images of *I*_φ_/*I*_φ + 120_ and *I*_φ_/*I*_φ_ _– 120_ by calculating the intensity ratio in each pixel of the images, which were further converted to a molecular orientation image using the relation in Figure 4c. As shown in Figure 6a, we successfully obtained the crystal orientation image. The orientation angles were almost the same across the island, i.e., approximately 94.8° from the angle φ. From this result, we illustrated the arrangement of DPh-DNTT molecules forming a herringbone structure in the island in Figure 6b. The *a*-axis direction almost matched the direction of the short axis, which was in good agreement with our previous study.^24^ This indicated that our method based on polarization Raman imaging worked consistently to image the crystal orientation distribution in the monolayer DPh-DNTT islands at a high spatial resolution of a few hundred nanometers.

Although we confirmed that the polarization Raman imaging technique is useful for analyzing the crystal orientation of monolayer DPh-DNTT islands, we believe that this technique holds the potential to gain a much higher analytical ability through further developments. As our aim is to develop a technique to study the initial stage of organic film growth, we developed this technique for investigation of an organic monolayer, and thus it is not appropriate to evaluate organic films having more than a monolayer with the current analytical algorithm. By further improving the algorithm to simultaneously address multiple different crystal orientations that exist within a multilayered organic film, it would be possible to extend the analytical ability of this technique for thick samples. In addition, although it is easy to accurately analyze the crystal orientation on a flat substrate such as the oxidized Si substrate, it can be complex if the substrate is not smooth. The focal depth of the incident laser is ∼1 μm in our setup. The maximum imaging area is 20 μm × 20 μm so far, as shown in Figure 3. By further improving the experimental setup and the analytical algorithm, our technique can be applied for samples on a rough substrate or for large-area imaging.

In this study, we assumed that DPh-DNTT molecules stand vertically to the substrate, but there may be a slight tilt from the substrate. By applying radial polarization, a polarization perpendicular to the substrate can be created, which could be effectively used to evaluate the tilt of organic semiconducting molecules on the substrate. Such a molecular tilt analysis was successfully demonstrated with pentacene molecules,^41,42^ where the polarization component perpendicular to the substrate could be further enhanced by employing the high-NA parabolic mirror.^43^ By combining this method with the technique we developed in this study, our technique could be extended to study the three-dimensional orientation of DPh-DNTT molecules. Additionally, as the Raman spectrum itself contains chemical information, the multimodal analysis would provide more details about organic semiconducting samples by correlating molecular orientation with the information of chemical bonds. Particularly, the low-frequency region of the Raman spectrum contains intermolecular interaction information,^44−46^ which could give important insights into how densely DPh-DNTT molecules are packed in the herringbone structure. Furthermore, if a higher spatial resolution is required in a crystal orientation image, tip-enhanced Raman spectroscopy could be utilized.^36,43,47−52^ Although the polarization control of a near-field light is still challenging, a technique based on a defocused imaging technique to evaluate and control the polarization of near-field light at a metallic tip apex has recently been demonstrated.^51,52^

## 3 Conclusions

Overall, we demonstrated crystal orientation analysis of a monolayer DPh-DNTT island using polarization Raman microscopy. We developed a method to determine the crystal orientation of the island utilizing the relationship between Raman intensity and the polarization direction of the incident light. Upon applying it to a cruciform island, we confirmed our method’s validity and demonstrated crystal orientation mapping at a high spatial resolution of approximately 300 nm. This spatially resolved, high-resolution imaging method could be beneficial not only for DPh-DNTT but also for various DNTT derivatives.

## 4 Experimental Section

### 4.1 Preparation of DPh-DNTT Samples

Si substrates with thermally grown 90 nm thick SiO_2_ were used. After the substrate was cleaned in acetone and isopropanol with an ultrasonic cleaner, the surface of the substrate was treated with UV–O_3_ irradiation for 30 min or chemical etching with HF solution.^53^ In the latter process, the substrate was immersed in HF solution diluted to 2.5 vol % with deionized water for 30 s, which etches the SiO_2_ surface by a few nanometers. A DPh-DNTT submonolayer film was deposited on the substrate at a pressure on the order of 10^–4^ Pa with a deposition rate of 0.05 Å/s. The shape of the DPh-DNTT islands can be controlled by the substrate temperature during the deposition and/or the surface treatment. The round islands were formed at 160 °C on the substrate treated with UV–O_3_. Meanwhile, the cruciform islands were formed at 185 °C on a substrate treated with the HF solution. DPh-DNTT was supplied by Nippon Kayaku Co., Ltd.

### 4.2 Optical Setup for Raman Measurements

A single-mode laser (Torus 532, Laser Quantum) was used for excitation of Raman scattering. The laser was guided to an inverted optical microscope (ECLIPSE Ti, Nikon) after a beam expander. A highly stable sample stage (KS-N, Nikon) and a piezo scanner (BIO2.200, Piezoconcept) were mounted on the microscope. A sample was placed on the sample stage, which was illuminated by the incident laser through a high-NA objective (NA 0.95, 150×). The incident polarization was controlled by a half-wave plate and a polarizer in the incident optical path. The scattered Raman signals were collected through the same objective with a backscattering configuration. The scattered signals passed through a depolarizer and a notch filter in the detection optical path and were introduced to a spectrometer (IsoPlane 160, Teledyne). The signals were finally detected by a highly sensitive Peltier-cooled CCD camera (PIXIS:100BRX, Teledyne).

## Supporting Information Available

AFM images of a round monolayer DPh-DNTT island and a cruciform monolayer DPh-DNTT island; analysis of sample damage by laser irradiation; Lorentzian curve fitting to the Raman spectrum; illustrations of vibration modes of DPh-DNTT; and detailed explanations of equations for crystal orientation analysis (PDF)
