# Supplementary material for: Polarization Raman Imaging of Organic Monolayer Islands for Crystal Orientation Analysis
Source: ACS Omega. 2021 Mar 31;6(14):9520–7. doi: 10.1021/acsomega.0c06313 (PMC8047675; doi:10.1021/acsomega.0c06313)
Supplement: Supplementary file 1 — ao0c06313_si_001.pdf [file ao0c06313_si_001.pdf]

Supporting Information

Polarization Raman Imaging of Organic Monolayer Islands for Crystal Orientation Analysis

Toki Moriyama[1], Takayuki Umakoshi[1,2]*, Yoshiaki Hattori[3]*, Koki Taguchi[1], Prabhat Verma[1], and Masatoshi Kitamura[3]

1. Department of Applied Physics, Osaka University, 2-1, Yamadaoka, Suita 565-0871, Japan

2. PRESTO, Japan Science and Technology Agency, 4-1-8 Honcho, Kawaguchi, Saitama, 332-0012, Japan

3. Department of Electrical and Electronic Engineering, Kobe University, 1-1, Rokkodai-cho, Nada, Kobe 657-8501, Japan

S1. AFM images of a round DPh-DNTT island and a cruciform DPh-DNTT island

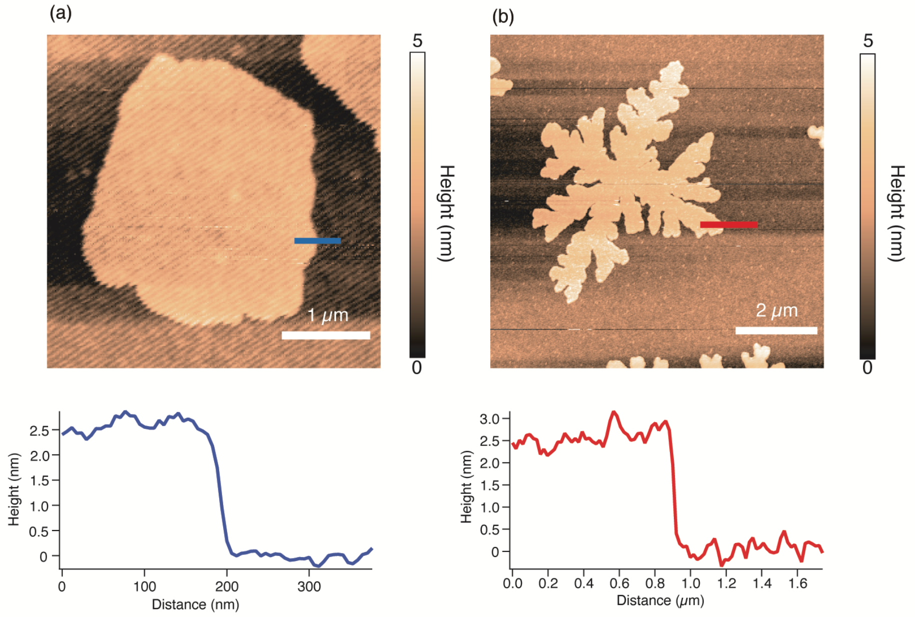

Figure S1. AFM images and topographic line-profiles of a round-shaped DPh-DNTT island (a) and a cruciform-shaped DPh-DNTT island (b).

S2. Stability of DPh-DNTT islands

We investigated the stability of our DPh-DNTT sample during Raman measurements. We kept illuminating the same spot on a round DPh-DNTT island with the incident laser for 400 s, and measured Raman spectra at various time intervals, as shown in Figure S2. The incident power was 240 μW, which is the same power as the one used for Raman imaging in Figure 3. In Figure S2a, we investigated the stability of the sample with an incident wavelength of 442 nm, while we used a wavelength of 532 nm in Figure S2b. As DPh-DNTT is expected to have a bandgap at around 450 nm,[1-3] we observed strong photoluminescence in Figure S2a, where incident wavelength was in near-resonance condition. As one can see, the observed scattering intensity gradually decreased with time. While we could observe weak Raman peaks ridding on a broad photoluminescence spectrum at 0 s, they quickly disappeared after 100 s. This confirms degradation of optical properties of the sample with time as both photoluminescence and Raman scattering decreased with time. In near-resonance condition, large number of photons are absorbed by the sample, resulting in a possible heating and a prominent photodegradation of the sample. Therefore, Raman measurements in near-resonance condition is not suitable for our sample for two reasons – one, a strong photoluminescence background appears that overwhelms Raman signal, and two, even weak illumination causes photodegradation.

In contrast, in the case of 532 nm in Figure S2b, Raman intensity at 1,395 cm^{-1}, which is the Raman mode used for the orientation analysis, was almost constant over 400 s. This clearly indicates that the sample damage due to laser irradiation at 532 nm, which is an off-resonance wavelength for our sample, was almost negligible.

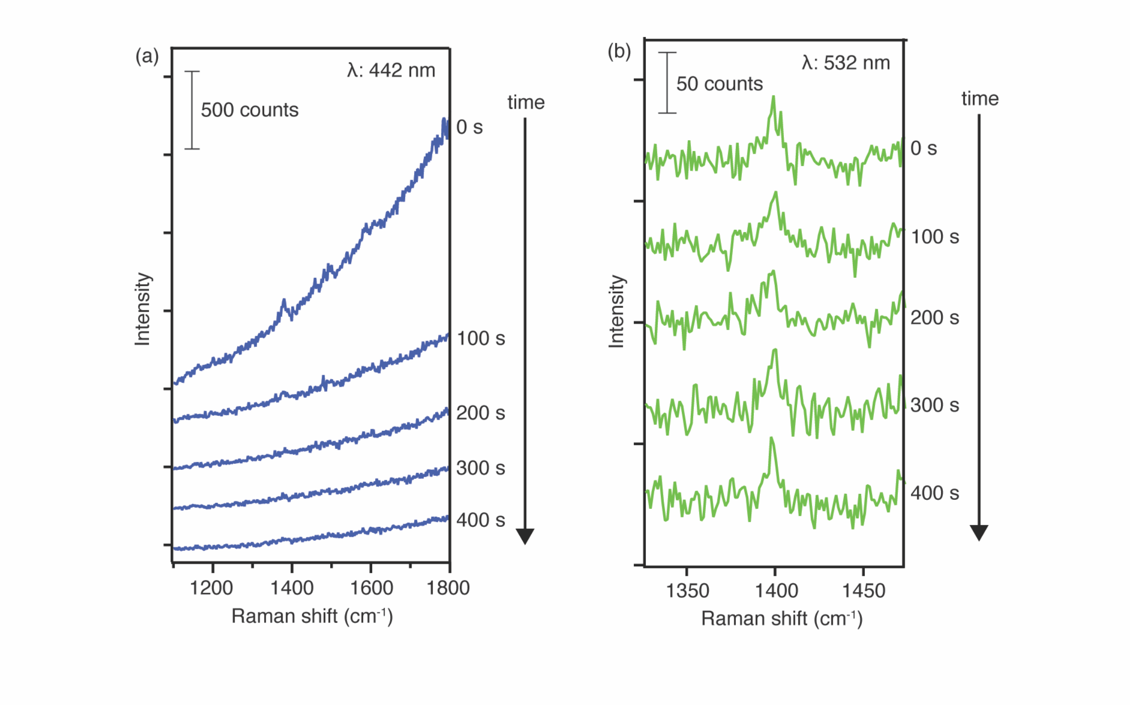

Figure S2. Raman spectra obtained from a round DPh-DNTT island with an acquisition time of 2 s for each spectrum at different time intervals under a continuous illumination of incident laser with a power of 240 μW. The incident wavelength was 442 nm in (a) and 532 nm in (b).

S3. Lorentzian curve fitting to Raman spectrum

We applied Lorentzian curve fitting to the Raman peak at 1,395 cm^{-1} to evaluate Raman intensity. Lorentzian function $f(\omega)$ is described as

$$f(\omega) = Y_0 + \frac{Aw^2}{4(\omega - \omega_0)^2 + w^2}.$$

Here, A is the peak height, *i.e.* Raman intensity, and w is the peak width. ω_0 is the peak position, and Y_0 is the base line of the spectrum. ω indicates Raman shift.

Figure S3 shows the Raman spectrum from Fig. 4a in the main text. This spectrum was obtained at θ=0°. We performed the curve fitting for this spectrum by using the above function, which is indicated by the blue curve. The spectral range used for the fitting was 1,360 to 1,440 cm^{-1}. Within this range, the background is almost a flat line (shown by green), and thus the background spectrum is expressed as the base line Y_0. For this spectrum, we obtained A = 239.9, w = 9.9, ω_0 = 1399.5, and Y_0 = 1410.7 from the best fitting, as indicated in Fig. S3.

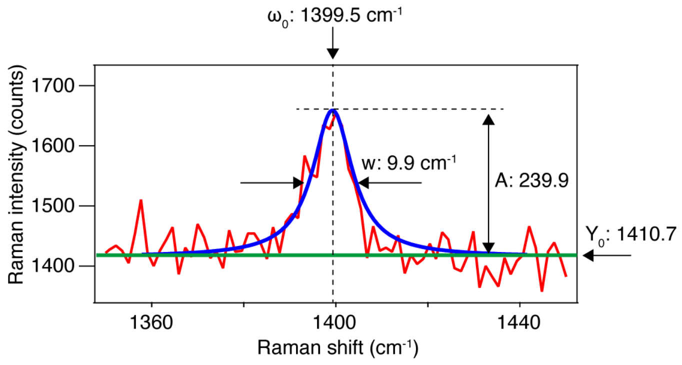

Figure S3. Raman spectrum taken from Fig. 4a in the main text, which was obtained at θ=0°. The blue curve indicates the Lorentzian-fitted curve.

S4. Successive Raman imaging of a cruciform DPh-DNTT island to investigate its stability

We further investigated the sample stability during Raman imaging. We performed Raman imaging of a cruciform DPh-DNTT island twice, where the second image was measured soon after the first one as a sequential measurement. The incident wavelength was 532 nm and the incident power was 420 μW, which are the same as those used for Raman imaging in Figure 5. The other experimental conditions are also the same as those used in Figure 5. The two images are shown in Figure S4, which look very similar to each other and there is no noticeable difference in Raman intensity between the two. If there was any sample damage during Raman imaging, the intensity of Raman image obtained in the second measurement should have been decreased. However, the intensities in both Raman images are almost same, which clearly indicates that there was no noticeable sample damage in our experiments.

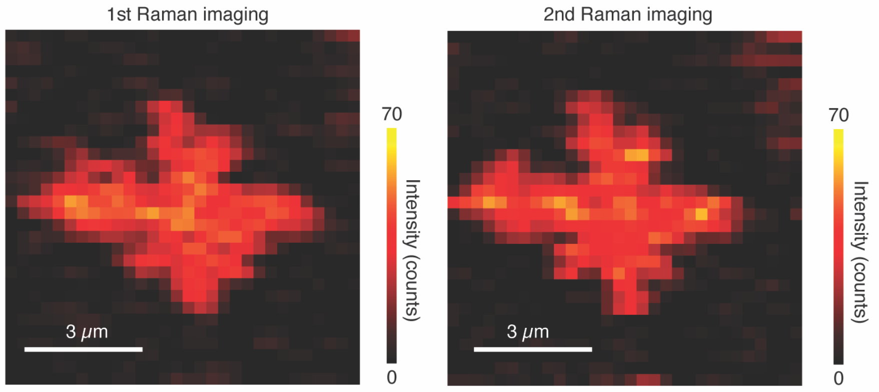

Figure S4. Raman images of a cruciform DPh-DNTT monolayer, where we performed Raman imaging twice to the same DPh-DNTT monolayer. No clear difference between the images was observed. The incident power was 420 μW, and the incident wavelength was 532 nm.

S5. Vibration mode of DPh-DNTT calculated by density function theory (DFT)

The in-plane atomic vibrations for a C-H bending mode and a C-C stretching mode of DPh-DNTT have been previously simulated through DFT calculations.[4] The vibrational frequencies of these two modes appear at 1416 cm^{-1} and 1427 cm^{-1}, respectively, the atomic vibrations of which are illustrated in Figure S5. The directions and the lengths of red arrows in Figure S5 indicate the direction of the vibrations and their corresponding strengths, respectively. The prominent Raman mode at 1395 cm^{-1} observed in Figure 1d can be assigned to either one of these two modes or to a combination of the two. A slight difference in the vibrational frequency between the calculation and experiment is due to the fact that the calculation was done for an isolated molecule under ideal conditions.

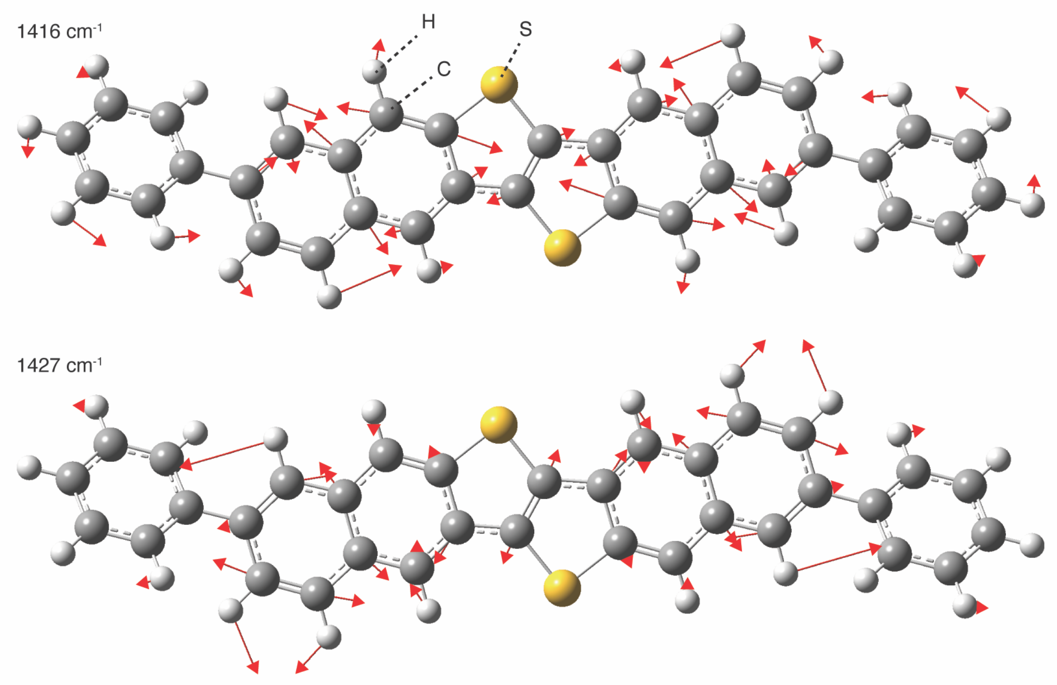

Figure S5. Schematics of vibration modes at 1,416 and 1,427 cm^{-1}, simulated through DFT method.

S6. Relationship between Raman intensity ratios and crystal orientation

The fitting curves in Figures 4a and S6a are expressed as $I(\theta) = A + B \cos^2\theta$, where $I(\theta)$ is Raman intensity at incident polarization angle θ; and the constants A and B are estimated from the best fitting as A = 27.2 and B = 128.6. Here, θ = 0 is defined as the angle where Raman intensity was the highest, which means θ = 0 corresponds to a direction parallel to the crystal orientation.

Next, to generalize this equation, consider the case where the crystal orientation angle is ψ, and the incident polarization angle is φ. We can obtain Raman intensity $I_\varphi(\varphi - \psi)$ as:

$$I_\varphi(\varphi - \psi) = C\,\{27.2 + 128.6 \cos^2(\varphi - \psi)\}.$$

Here, C is a coefficient that depends upon experimental conditions such as the laser power, the acquisition time of Raman signal or the optical alignment. In the case when the incident polarization angle matches the crystal orientation, *i.e.*, φ – ψ = 0, the highest intensity is obtained. When we plot this as a function of (φ – ψ), which means the incident polarization angle with respect to the crystal orientation, we obtain the black curve in Figure S6b. Similarly, $I_{\varphi+120}(\varphi, \psi)$ and $I_{\varphi-120}(\varphi, \psi)$ are expressed as:

$$I_{\varphi+120}(\varphi - \psi) = C\,[27.2 + 128.6 \cos^2\{(\varphi + 120) - \psi\}],$$

and

$$I_{\varphi-120}(\varphi - \psi) = C\,[27.2 + 128.6 \cos^2\{(\varphi - 120) - \psi\}],$$

which are shown in Figure S6b as the blue and the red curves, respectively. From the equations above and Figure S6b, the intensity ratios of $I_\varphi/I_{\varphi+120}$ and $I_\varphi/I_{\varphi-120}$ are expressed as:

$$I_\varphi/I_{\varphi+120} = [27.2 + 128.6 \cos^2(\varphi - \psi)] / [27.2 + 128.6 \cos^2\{(\varphi + 120) - \psi\}],$$

and

$$I_\varphi/I_{\varphi-120} = [27.2 + 128.6 \cos^2(\varphi - \psi)] / [27.2 + 128.6 \cos^2\{(\varphi - 120) - \psi\}].$$

In the process of taking the ratios, the coefficient C cancels out. When we plot these intensity ratios as functions of (φ – ψ), Figure S6c is obtained. We would like to consider the red curve ($I_\varphi/I_{\varphi-120}$) as an example. When (φ – ψ) ≈ 30°, $I_{\varphi-120}$ reaches its minimum, as seen in Figure S6b. Therefore, the intensity ratio $I_\varphi/I_{\varphi-120}$ achieves its maximum value at (φ – ψ) ≈ 30° in Figure S6c.

In contrast, when ($\varphi - \psi$) ≈ 90°, I_φ reaches its minimum, and thus $I_\varphi/I_{\varphi\text{-}120}$ takes the minimum value, as seen in Figure S6c. In this way, the relation between the intensity ratios and the incident polarization angle from the crystal orientation angle is obtained.

For the convenience of understanding the crystal orientation angle of the sample, instead of plotting the intensity ratio with respect to the incident polarization angle from the crystal orientation, *i.e.* ($\varphi - \psi$), as displayed in Figure S6c, it is reasonable to consider the orientation angle ψ with respect to a certain incident polarization angle φ, which means ($\psi - \varphi$). Therefore, in Figure 4c in the main text, the lateral axis is reversed from Figure S6c, and the curves are plotted as function of the orientation angle from a certain incident polarization angle, *i.e.* ($\psi - \varphi$).

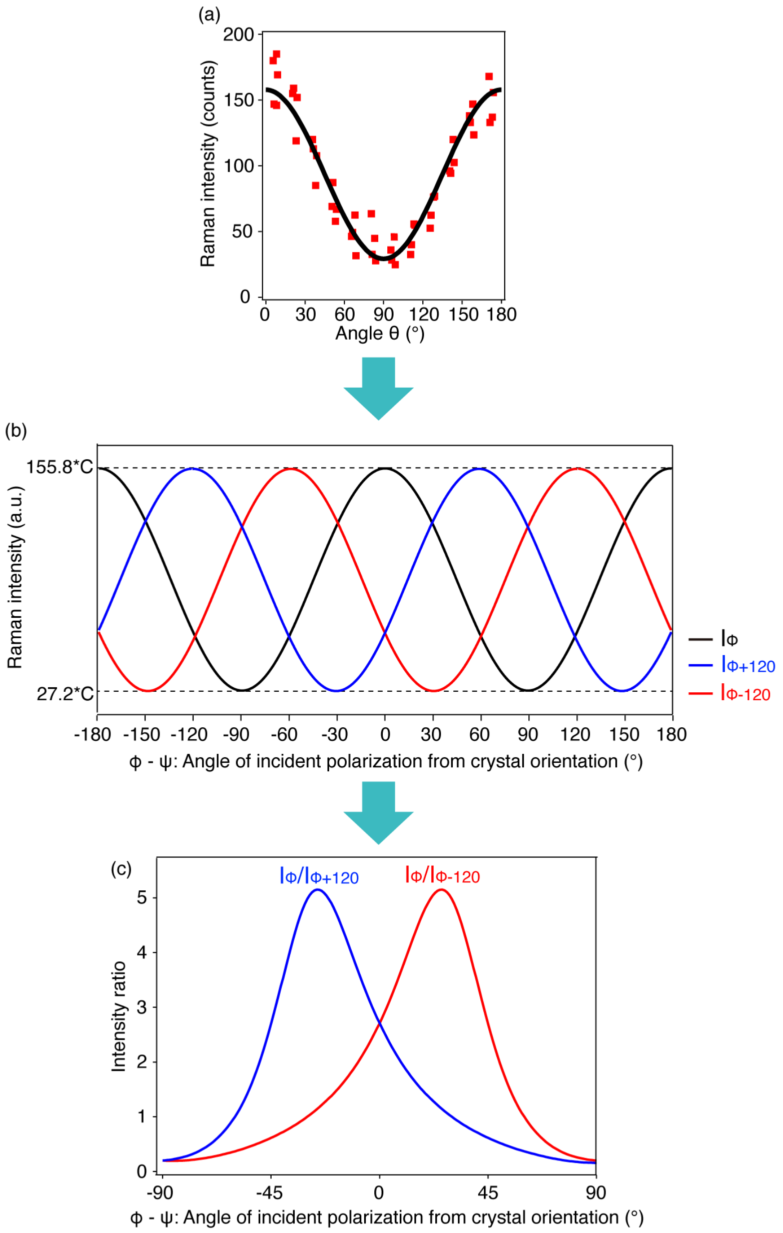

Figure S6. (a) Dependence of Raman intensity $I(\theta)$ on the incident polarization angle θ, where θ = 0 corresponds to the angle at which the highest intensity is obtained. The black line corresponds to a fitting curve using the equation $I(\theta) = A + B \cos^2\theta$, where the values of the constants A and B are obtained from the best fitting as $A = 27.2$ and $B = 128.6$. (b) Fitting curves of Raman intensities I_{φ}, $I_{\varphi+120}$, and $I_{\varphi\text{-}120}$ at different incident polarization angles φ, φ+120 and φ-120, respectively, which are plotted as functions of the incident polarization angle with respect to the crystal orientation angle, *i.e.* φ – ψ. (c) Dependence of the intensity ratios $I_{\varphi}/I_{\varphi+120}$ and $I_{\varphi}/I_{\varphi\text{-}120}$ on the incident polarization angle with respect to the crystal orientation angle (φ – ψ).

Reference

(1) Tanaka, S.; Miyata, K.; Sugimoto, T.; Watanabe, K.; Uemura, T.; Takeya, J.; Matsumoto, Y. Enhancement of the Exciton Coherence Size in Organic Semiconductor by Alkyl Chain Substitution. *J. Phys. Chem. C* **2016**, *120* (15), 7941–7948.

(2) Kang, M. J.; Doi, I.; Mori, H.; Miyazaki, E.; Takimiya, K.; Ikeda, M.; Kuwabara, H. Alkylated Dinaphtho[2,3-b:2′,3′-f]Thieno[3,2-b]Thiophenes (Cn-DNTTs): Organic Semiconductors for High-Performance Thin-Film Transistors. *Adv. Mater.* **2011**, *23* (10), 1222–1225.

(3) Sawamoto, M.; Kang, M. J.; Miyazaki, E.; Sugino, H.; Osaka, I.; Takimiya, K. Soluble Dinaphtho[2,3-b:2′,3′-f]Thieno[3,2-b]Thiophene Derivatives for Solution-Processed Organic Field-Effect Transistors. *ACS Appl. Mater. Interfaces* **2016**, *8* (6), 3810–3824.

(4) Hattori, Y.; Kimura, Y.; Yoshioka, T.; Kitamura, M. Data on Optical Microscopy and Vibrational Modes in Diphenyl Dinaphthothienothiophene Thin Films. *Data Br.* **2019**, *26*, 104522.
